# Paroxetine alleviates T lymphocyte activation and infiltration to joints of collagen-induced arthritis

**DOI:** 10.1038/srep45364

**Published:** 2017-03-28

**Authors:** Qingtong Wang, Longsheng Wang, Li Wu, Mei Zhang, Shanshan Hu, Rui Wang, Yongsheng Han, Yujing Wu, Lingling Zhang, Xinming Wang, Wuyi Sun, Wei Wei

**Affiliations:** 1 Institute of Clinical Pharmacology, Anhui Medical University, Key Laboratory of Anti-inflammatory and Immune Medicine, Ministry of Education, Collaborative Innovation Center of Anti-inflammatory and Immune Medicine, Hefei, 230032, China; 2 Emergency Center, Affiliated Anhui Provincial Hospital, Anhui Medical University, Hefei, China

## Abstract

T cell infiltration to synovial tissue is an early pathogenic mechanism of rheumatoid arthritis (RA). In the present work, we reveal that G protein coupled receptor kinase 2 (GRK2) is abundantly expressed in T cells of collagen-induced arthritis (CIA). A GRK2 inhibitor, paroxetine protects the joints from inflammation and destruction, primarily through inhibition of both CD4^+^ helper T (Th) cell and CD8^+^ cytotoxic T (Tc) cell migration to synovial tissue. Meanwhile, paroxetine restores the balance of Th/Tc, effector Th (Theff)/ naïve Th (Thnaive) and effector Tc (Tceff)/ naïve Tc (Tcnaive) to equilibrium by elevating the frequency of Thnaive, Tcnaive and regulatory Th cells; reducing the increased Theff, activated Th and Tceff, having a similar effect as methotrexate (MTX). In addition, both serum and synovial IL-1β, TNF-α and CX3CL1 expression was effectively inhibited in treated rats. *In vitro* assay confirmed that paroxetine inhibits CX3CL1-induced T cell migration through blocking the activity of GRK2. Among three MAPK families, paroxetine was found to be able to decrease the phosphorylation of ERK. This study elucidates that paroxetine attenuates the symptoms of CIA rats due to its inhibitory effect on T cell activation and infiltration to synovial tissue via suppression of ERK pathway.

Rheumatoid arthritis (RA) is a common chronic autoimmune disease with unknown etiology. The primary pathological process of RA occurs following the aberrant activation of the immune system, then abundant cytokines and chemokines are secreted, inducing immunocyte infiltration in synovium. This leads to synoviocyte dysplasia, matrix metallo proteinases (MMPs) production, and osteoclast differentiation, which results in bone destruction and functional incapacitation of facet joints^1^. Glucocorticoid and non-steroidal anti-inflammatory drugs (NSAIDs) are used to relieve the acute joint swelling and pain; disease-modifying drugs (such as methotrexate, MTX) and biologics (like TNF-α inhibitors) are widely used in treating established RA patients^2^,^3^,^4^. However, this disease cannot be completely cured. Developing new effective drugs or discovering new therapeutic targets is extremely urgent.

Immunocytes including macrophages, dentritic cells, B cells, and particularly T cells migrate to the synovial tissue under the interaction of chemokines and chemokine receptors. Of note, the accumulation of immune cells in synovium is the promoter of local joint inflammation^5^. Therefore, attenuating chemokine receptor signaling and preventing lymphocytes migration could be a promising therapeutic strategy for RA. Among the various chemokines, CX3C chemokine ligand 1 (CX3CL1) is regarded as a pivotal T cell chemoattractant. It is overproduced in T cells and attracts T cells to synovium by interacting with its receptor CX3C chemokine receptor 1 (CX3CR1) which is expressed on fibroblast-like synoviocytes (FLSs)^6^. The signaling pathway of CX3CR1, a G protein coupled receptor (GPCR), potently controls the migration of T cells^7^. Furthermore, CX3CR1 function is precisely regulated by G protein coupled receptor kinases (GRKs).

Among the seven GRKs subtypes, GRK2 is emerging as the pivotal integrative scaffold for cell motility including in epithelial cells and fibroblast cells^8^,^9^. Accumulating data indicate the dysfunction and overexpression of GRK2 in RA patients and animal models, suggesting that GRK2 could be a promising target of RA treatment^10^. However, there is no GRK2 specific inhibitor available in the market. Fortunately, paroxetine, a selective serotonin reuptake inhibitor commonly prescribed as an antidepressant was found to have GRK2 inhibitory ability with IC50 is 35 μM^11^,^12^. We therefore hypothesize that paroxetine treatment would inhibit the T cell infiltration to the synovial tissue of rats with collagen-induced arthritis (CIA) and therefore attenuate the synovitis. Our data will demonstrate for the first time the therapeutic effect of paroxetine on RA and its potential mechanisms.

## Results

### Paroxetine treatment attenuates the symptoms of CIA rats

**Figure 1:**
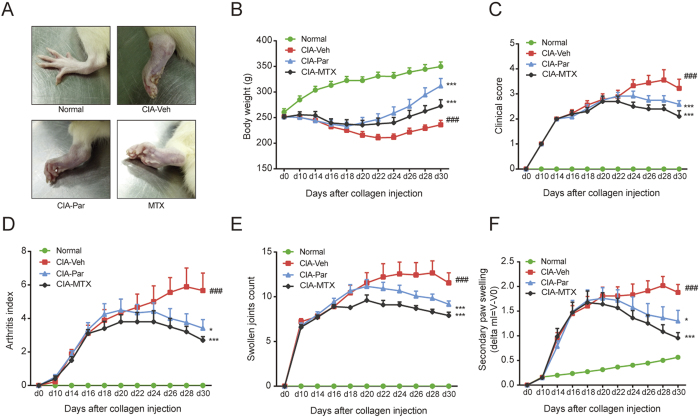
Paroxetine treatment obviously attenuated the symptoms of CIA rats. Wistar rats were immunized with chicken type II collagen to establish CIA model. The onset of arthritis was on around d14 after the first injection. CIA rats were randomly divided into different groups according to the global assessment. The representative pictures of swelling joints were presented (**A**). Body weight (**B**) was measured every 3 days. Clinical score (**C**), arthritis index(**D**), swollen joints count (**E**) were evaluated following the published standard. Secondary paw swelling (**F**) was checked with a toe volume meter and calculated by subtracting the paw volume at d0. Data are mean ± SD of ten rats. ^*###*^*p* < 0.001 versus normal group; *p < 0.05, ***p < 0.001 versus CIA-Veh group.

An emulsion of collagen was injected into the right hindpaw to induce CIA, which cause primary inflammation within 24–48 h from the first immunization. Body weight and pathologic manifestations were observed every three days after the booster injection. The secondary inflammation, which is caused by a systemic autoimmune response, developed on or around day 14. The fur of model rats lost luster and looked dull, their movement was limited due to the inflammation of their joints (Fig. 1A). CIA rats showed a severe body weight loss, with significant higher scores in clinical manifestation, arthritis index, the number of swollen joints, and size of secondary paw swelling (Fig. 1B–F). Both paroxetine and the widely used RA treatment of MTX clearly have positive effects on attenuating the symptoms of arthritis in rats. Of note, paroxetine treatment helped CIA rats to restore more body weight even though there is no significant difference comparing with MTX group. On alleviating the global assessment of CIA rats, paroxetine administration exerted moderate effects compared with that of MTX.

### Paroxetine treatment markably alleviates T cell infiltration into synovial tissue

**Figure 2:**
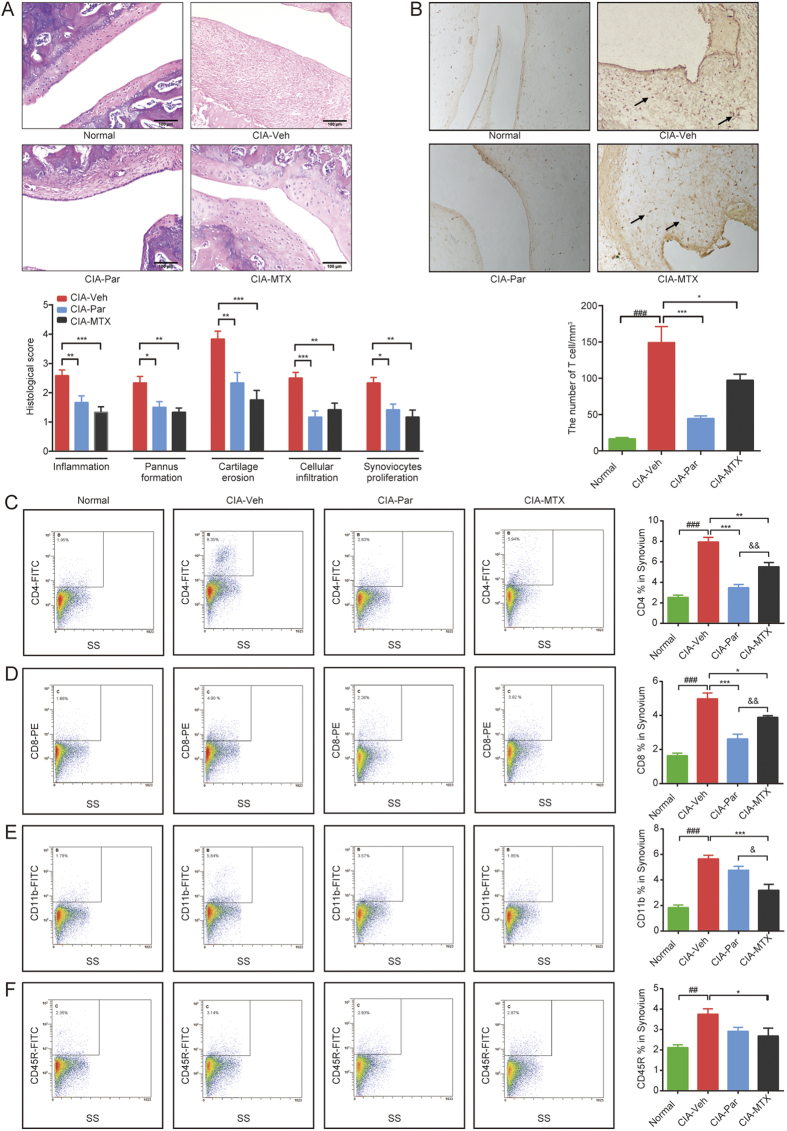
Paroxetine treatment remarkably alleviated T cells infiltration into synovial tissue. After the treatment, the H&E staining of the joints was performed, pathogenic parameters (**A**) including inflammation, pannus formation, cartilage erosion, cellular infiltration and synoviocytes proliferation was evaluated. T cells in the synovial tissue were displayed by staining CD3 via immunochemistry, and T cell numbers were counted using image pro-plus (**B**). The single cell suspension of synovial tissues were made by enzymatic isolation method. The frenquency of CD4^+^ T cells (**C**), CD8^+^ T cells (**D**), CD45R^+^ B cells (**E**) and CD11b+ macrophages (**F**) were detected by flow cytometry. Data are mean ± SD of five rats. ^*##*^*p* < 0.01, ^*###*^*p* < 0.001; *p < 0.05, **p < 0.01, ***p < 0.001; ^*&*^*p* < 0.05, ^*&&*^*p* < 0.01.

We investigated the severe pathological changes of CIA joints revealed by H&E staining. The normal arthrosis has only 1 to 3 layers of aligned synoviocytes, however, histological examination of CIA joints showed an excessive and disorganized proliferation of synoviocytes, with large amounts of lymphocyte infiltration, abundant pannus formation, obvious cartilage erosion and distinct local inflammation (Fig. 2A). As expected, MTX significantly alleviated the synovium inflammation and bone destruction of CIA joints. Paroxetine treatment clearly prevented the histological damage of joints in CIA rats as well. Notably, paroxetine showed a better inhibitory effect on immune cell infiltration than MTX treatment. Previous evidence showed that the majority of the infiltrated immunocytes are T cells^13^, therefore, we investigated the effect of paroxetine on immunocytes especially on T cell migration to CIA synoviums.

CD3 is the surface marker for indentifying pan-T cells. We stained the joint slides with anti-CD3 antibody to investigate the extent of T cell infiltration in joints of different treatment groups. Immunohistochemical assay found a large amount of T cells were recruited into the synovial tissue of CIA arthrosis. Paroxetine treatment markedly reduced the number of T cells detected in the synovium. However, MTX was found to have a relatively weaker effect (Fig. 2B).

We then digested the synovial tissues with collagenase II and trypsin. The single cell suspension was analyzed using a flow cytometer after staining. Large numbers of CD4^+^ helper T (Th) cells, CD8^+^ cytotoxic T (Tc) cells, CD45R^+^ B cells and CD11b^+^ macrophages were found in synovial tissue suspensions of CIA rats (Fig. 2C–F). Paroxetine treatment substantially reduced the infiltration of Th and Tc cells into synovial tissue, but didn’t show obvious effects on attenuating the migration of B cells and macrophages. In accordance with the immunohistochemical determination, MTX exerted a moderate effect on inhibiting Th and Tc cells infiltration comparing with paroxetine, however, it strongly deterred the recruitment of macrophages and B cells. These data indicate that paroxetine has profound effect on preventing T cell infiltration which correlates with a relief of local inflammation and attenuation of the symptoms of CIA rats.

### Paroxetine treatment prevents Th cell differentiation and activation in rats with CIA

**Figure 3:**
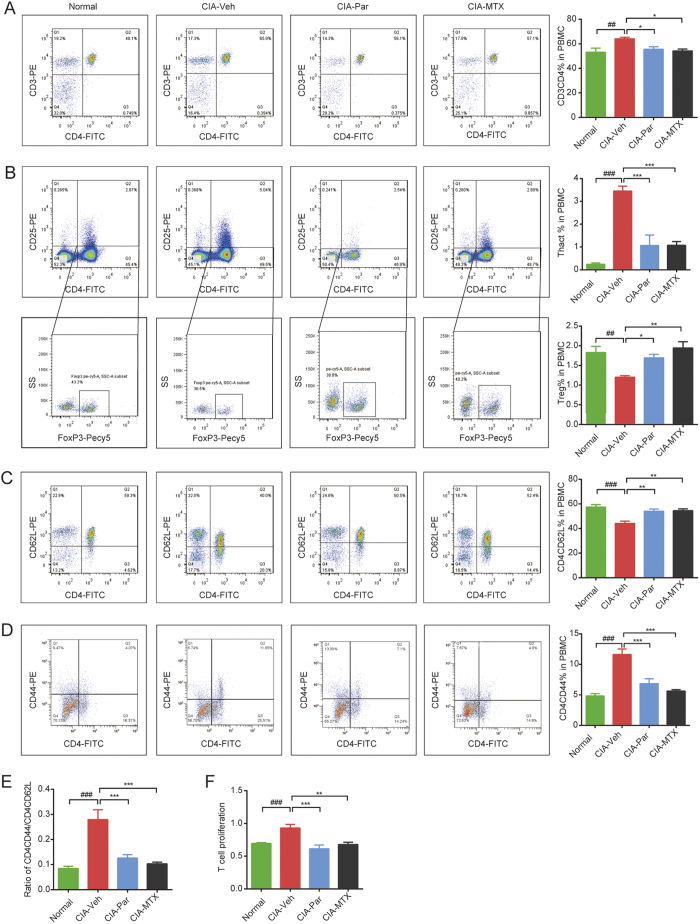
Paroxetine treatment prevented CD4^+^ T cells differentiation and activation in PBMC of rats with CIA. Monocytes were isolated from peripheral blood by density gradient centrifugation. The population of CD3^+^ CD4^+^ helper T cells (**A**), CD4^+^ CD25^+^ T cells (**B**), CD4^+^ CD25^+^ FoxP3^+^ Treg cells (**C**), CD4^+^ CD62L^+^ naïve T cells (**D**) were determined by flow cytometry. The percentage of effective T cells was calculated by subtracting the percentage of Treg cells from CD4 and CD25 DP T cells (**E**). The proliferation of splenic T cells purified from each group was detected by CCK assay (**F**). Data are mean ± SD of five rats. The proliferation experiment was conducted in triplicate.^*##*^*p* < 0.01, ^*###*^*p* < 0.001; *p < 0.05, **p < 0.01, ***p < 0.001.

Next, we explored weather paroxetine influences T cell activation. The analysis of flow cytometry with the peripheral blood mononuclear cells (PBMCs) from CIA rats showed a substantial increase in the percentage of CD3 and CD4 double positive (DP) T cells^14^,^15^, which are defined as helper T (Th) cells and facilitate the adaptive immune response by releasing various pro-inflammatory cytokines (Fig. 3A). Both CD4 positive and CD25 low expression T cells, as well as CD4 positive and CD25 high expression T cells are induced by antigen stimulation and the activation of T cell receptor (TCR) signaling. However, these two kinds of T cells have totally opposite functions. CD25 low expression T cells are named as activated T cells (Thact) which promote the inflammatory immune response, while CD25 high expression Th cells defined as regulatory Th cells (Threg) are involved in inhibiting the immune system and retaining immune tolerance^16^. To precisely distinguish these two subpopulations, we stained CD4, CD25 and foxp3, a recognized transcription factor for analyzing Threg cells. The percentage of Thact cells was calculated by subtracting the percentage of Threg cells from CD4 and CD25 DP T cells. As a result, the frequency of Thact were obviously elevated, while Threg cells were significantly reduced (Fig. 3B) in the established CIA suggesting that the autoimmune responses were over-activated. Meanwhile, naïve CD4 and CD62L DP (Thnaive) cells were found to be decreased but CD4^+^CD44^+^ effector Th (Theff) cells were elevated in PBMC of CIA rats (Fig. 3C and D) with an increased ratio of Theff/Thnaive (Fig. 3E). The administration of paroxetine turned out to have similar effects as MTX in reducing the Th, Thact and Theff subsets, maintaining the CD4^+^ naïve and Treg subgroups.

CCK assay was applied to detect the vitality and proliferation of splenic T cells. ConA was used as a stimulator. As expected, T cells from CIA rats proliferated notably, and were found to form multiple colonies when observed with a microscope before adding CCK8. MTX substantially inhibited T cell proliferation. The GRK2 inhibitor, paroxetine, significantly reduced T cell vitality as well (Fig. 3F), indicating that it has an immune regulatory effect by modulating the differentiation, activation and proliferation of T cells.

### Paroxetine treatment prevents Tc cell activation in rats with CIA

**Figure 4:**
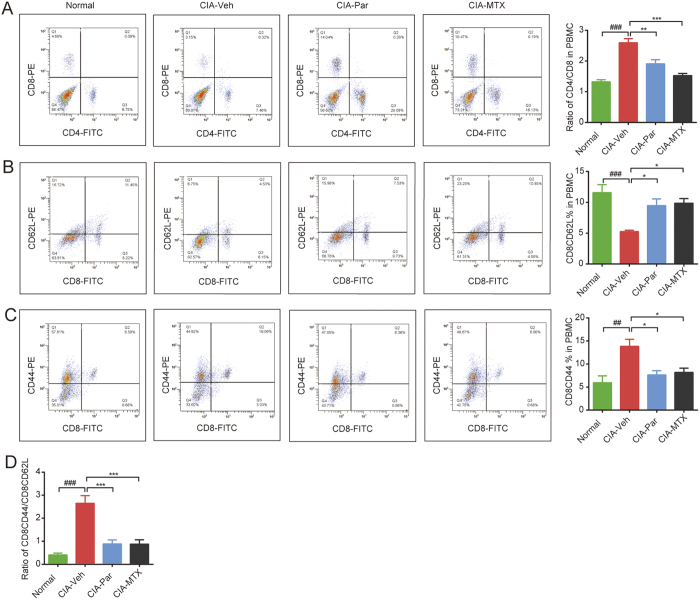
Paroxetine treatment prevented CD8^+^ T cells differentiation and activation in PBMC of rats with CIA. The ratio of CD4^+^ to CD8^+^ T cells (**A**), the frequency of CD8^+^ CD62L^+^ naïve Tc cells (**B**), CD4^+^ CD44^+^ effector Tc cells (**C**) were determined by flow cytometry. The ratio of effector Tc cells to naïve Tc cells was calculated (**D**). Data are mean ± SD of five rats. ^*##*^*p* < 0.01, ^*###*^*p* < 0.001; *p < 0.05, **p < 0.01, ***p < 0.001.

Similarly, we found that the population of Tc cells shrunk and the ratio of Th to Tc cells was increased accordingly under the pathological state (Fig. 4A). Meanwhile, the subset of CD8^+^CD44^+^ effector T (Tceff) cells was increased while the CD8^+^CD62L^+^ naïve T (Tcnaive) cells was reduced in PBMC of CIA rats (Fig. 4B and C), leading to an obviously elevated ratio of Tceff to Tcnaive (Fig. 4D). Both paroxtine and MTX treatment significantly suppressed the activation of Tc cells.

### Paroxetine treatment reduces cytokine and chemokine levels in serum and synovial tissues of CIA rats

**Figure 5:**
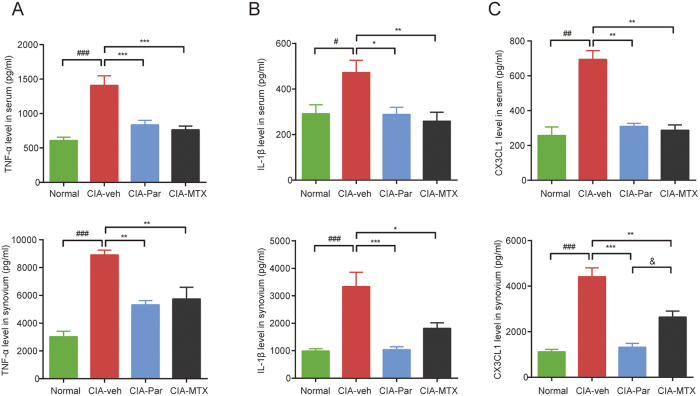
Paroxetine treatment reduced cytokines and chemokines levels in serum and synovial tissues of CIA rats. The homogenate of 15mg synovial tissue was made by an electric homogenizer with 200 μl lysis buffer containing proteinase inhibitors. Serum and synovial homogenate expression of TNF-α (**A**), IL-1β (**B**) and CX3 CL1 (**C**) were determined by ELISA. Data are mean ± SD of five rats, each sample and standard curve was detected in duplicate. ^*##*^*p* < 0.01, ^*###*^*p* < 0.001; *p < 0.05, **p < 0.01, ***p < 0.001.

Cytokine production is an important function of activated T cells. We detected the effect of paroxetine and MTX on the secretion of representative pro-inflammatory cytokines, like TNF-α and IL-1β in rats with CIA. Both TNF-α and IL-1β were abundantly expressed in serum and synovial tissue of CIA rats (Fig. 5A and B). Paroxetine and MTX showed positive effects on reducing the production of these cytokines. Paroxetine especially significantly decreased the local levels of TNF-α and IL-1β in synovium, which is in accordance with our evidence showing that paroxetine distinctly prevents the T cell infiltration into the synovial tissue and inhibits the T cell differentiation and activation, therefore suppressing their secretory capacity.

As we known, CX3CL1 is one of the pivotal chemokines involved in T cell migration^17^. In the serum and synovial homogenate of CIA rats, we detected much higher concentrations of CX3CL1 than that in normal rats (Fig. 5C). The level of CX3CL1 is reported to be induced by TNF-α and other pro-inflammatory cytokines^17^. Both paroxetine and MTX could reduce the level of CX3CL1 in serum and synovial tissues but with different extent. Paroxetine revealed a stronger effect than MTX on inhibiting CX3CL1 production in synovial tissues. These data illustrates that the anti-arthritis effect of paroxetine is associated with the suppression of T cell activation, reduction of chemokine secretion, and the inhibition of T cell infiltration to joints.

### Paroxetine distinctly restrains T cell migration induced by CX3CL1 through inhibiting GRK2

**Figure 6:**
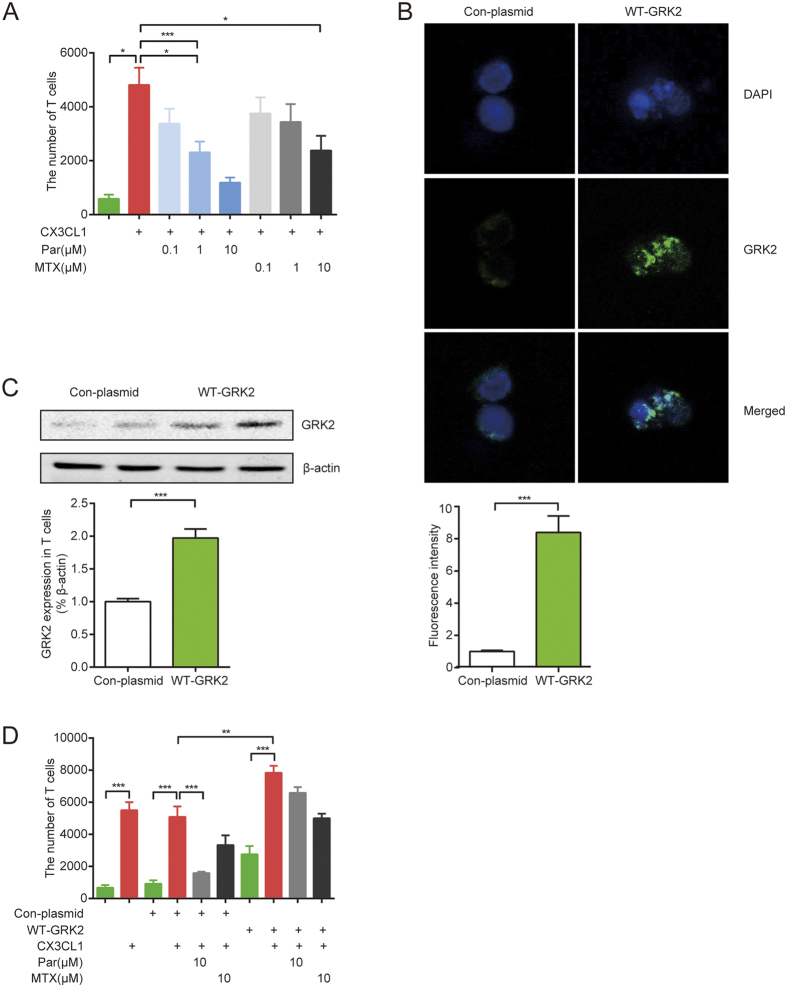
Paroxetine distinctly restrainted T cell migration induced by CX3CL1 through inhibiting GRK2. The *in vitro* T cell migration assay was performed using a transwell system. 5 × 10^5^ splenic T cells were added on the top chamber and 10 μg/ml CX3CL1 was added on the bottom well. 0.1, 1, 10 μM paroxetine or 0.1, 1, 10 μM MTX was administrated into the system at the same time. (**A**) The number of T cells that migrated to the bottom well was counted. T cells were electrotransfected with pIRES2-EGFP-GRK2 or pIRES2-EGFP vector, the expression of GRK2 was detected by immunofluorescence assay (**B**) and western blot (**C**). Migration assay was performed again using T cell transfected with either WT-GRK2 plasmid or control-plasmid (**D**). Data are mean ± SD of four independent experiments, each condition was detected in duplicate. *p < 0.05, **p < 0.01, ***p < 0.001.

To confirm that paroxetine blocks CX3CL1-induced T cell migration, we conducted an *in vitro* T cell migration experiment. Serum free DMEM media with 10 μg/ml CX3CL1 was added into the lower chamber of the transwell plate, and 5 × 10^5^ splenic T cells purified by immunomagnetic beads were added into the upper chamber. 1 μM and 10 μM paroxetine pretreatment reduced the number of T cells migrating from the cell insert to the lower chamber. 1 μM MTX moderately inhibited T cell migration (Fig. 6A). To further investigate the role of GRK2 in this system, we transfected T cells with wild type GRK2 plasmid (GRK2-T cells) by electroporation, which greatly increased GRK2 expression (Fig. 6B transfection efficiency 59.2%). T cells overexpressing GRK2 had higher migration ability than the T cells transfected with control plasmid (Con-T cells). However, paroxetine could not successfully block the migration of GRK2-T cells, but inhibited the migration of Con-T cells (Fig. 6C). These data demonstrate that GRK2 indeed plays a positive role in T cells migration.

### Paroxetine inhibits GRK2 induced activation of ERK

**Figure 7:**
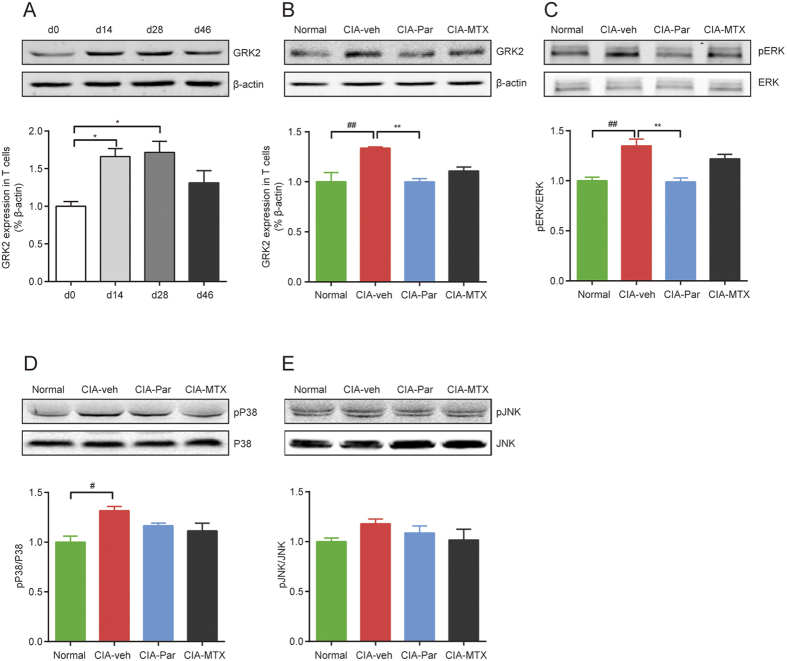
Paroxetine inhibited GRK2 induced activation of ERK. The expression of GRK2 in splenic T cells at different stages of CIA was detected (**A**), the onset of CIA is around d14 after the first immunization, the most severe stage is around d28, and the remission stage occurs after about 46 days. After the treatment, the expression of GRK2 in splenic T cells was determined again (**B**). The activation of MAPK family signaling pathway including ERK (**C**), p38-MAPK (**D**) and JNK (**E**) was detected by western blot. Data are mean ± SD of four rats. ^*#*^*p* < 0.05, ^*##*^*p* < 0.01; *p < 0.05, **p < 0.01.

In rats injected with chicken type II collagen (CCII),clinically apparent arthritis with swollen joints appears around 14 days after the first immunization. The most severe pathogenic manifestations appear on around d28, then slowly, the inflammation subsides on or around d46. Western blot data shows that the expression of GRK2 in splenic T cells was obviously increased with the onset of arthritis and was recovered in the remission period (Fig. 7A), suggesting that GRK2 is involved in the progress of RA. We also found that paroxetine treatment even could reduce the expression level of GRK2 besides the activity inhibition as reported (Fig. 7B), while MTX had no significant effect on GRK2 expression. Accumulating evidence revealed that the mitogen-activated protein kinases (MAPKs) pathway primarily controls T cell migration^13^. Three branches of Jun N-terminus kinase (JNK), p38 MAPK and ERK are the well-known downstream effectors of MAPK pathway. We detected the phosphorylation of each signaling molecule and found that p38 and ERK were markedly activated in T cells of CIA rats, while JNK was slightly activated. However, paroxetine only substantially blocked the activation of ERK (Fig. 7C–E). These results indicate that the inhibitory effect of paroxetine on T cell migration is mediated by the ERK pathway.

## Discussion

The increased expression of local chemokines secreted by resident cells in joints recruits various immunocytes including T cells, B cells and macrophages, etc. to the synovial tissue. Subsequently,the immune cells are activated, release a large amount of pro-inflammatory cytokines and induce the activation and proliferation of synoviocytes, and as a result, progressive joint inflammation is initiated^13^. Strategies on inhibiting immunocytes migration may efficiently stop the progression of RA in early stage, allow for patients to have a higher quality of life.

In the present work, we found abundant Tc cells, Th cells, B cells and macrophages were infiltrated to the synovial tissue of CIA rats. A inhibitor of GRK2, paroxetine exerted better effects on attenuating both Th and Tc cell migration and infiltration than that of MTX. Paroxetine only slightly inhibited the migration of macrophages, which also play an important role in joint inflammation. Abundant literature has reported that while CX3CL1 is significant for T cell migration, granulocyte-macrophage colony-stimulating factor (GM-CSF) is the key chemokine for survival, proliferation, differentiation, maturation and functional activation of hematopoietic cells, including macrophages and monocytes^18^,^19^. GM-CSF receptor is a heterodimer composed of α and β subunits^20^. A reduction of macrophages in synovial tissue has been correlated with improvement in the disease activity score, thus antagonizing GM-CSF was found to markedly reduce established disease in mouse models of RA^21^,^22^. In order to protect the synovial tissue from inflammatory cell infiltration, a synergistic effect will be expected with the combined treatment of paroxetine and GM-CSF inhibitor for RA patients. Intercellular cell adhesion molecule-1 (ICAM-1), vascular cell adhesion protein 1 (VCAM-1), and CXCL13 are involved in B cell migration^23^. As we known, receptor of GM-CSF, ICAM-1 and VCAM-1 are not belong to GPCRs. Therefore, paroxetine could not significantly inhibit the infiltration of macrophages and B cells. However, MTX exerted nonspecific inhibitory function on the migration of various immune cells.

According to the results of immunochemistry assay and flow cytometry, paroxetine notably reduced both Th and Tc cell migration to the synovium, partially due to it significantly decreased the synovial CX3CL1 secretion by resident cells. On the other hand, as reported by DeFord-Watts LM and his colleagues, GRK2 interacts with CD3 ε, maintains the activity of CD3 ε or basic-rich stretch (BRS) associated kinase, and regulates TCR signaling^24^. As we know there are three MAPK signalings arms of JNK, p38 MAPK and ERK, which we show here are activated in T cells from CIA rats at various degrees. All the three pathways are reported to be involved in medicating T cell migration^25^. ERK1/2 are important downstream molecules of TCR signaling, moreover, GRK2 *per se* is a scaffold protein for the activation of ERK pathway^26^. We observed that the increments of GRK2 expression and ERK1/2 activation in activated T cells were inhibited by paroxetine administration, which correlated with a decrease in differentiated T cell infiltration to the synovium. Therefore, we surmise that paroxetine prohibits the differentiation and migration of T cells probably through regulating TCR-ERK1/2 signaling.

MTX exerted its therapeutic effect through global immune suppression^27^, however paroxetine prevents the joint inflammation which is at the very early stage. As a consequence, paroxetine treatment might be less likely to cause harmful side effects such as gastrointestinal reaction and bone marrow suppression. *In vitro* paroxetine treatment observably inhibited the migration of T cells towards CX3CL1, this effect was blocked by overexpression of GRK2, fully established that elevated GRK2 expression contributes to T cell migration and GRK2 is the therapeutic target of paroxetine. Therefore, GRK2 will be a promising target for the strategy of treating early stage RA.

Previous findings have indicated that various GPCRs including formyl-peptide receptors, classical chemoattractant receptors and chemokine receptors are expressed on T cells and participate in T cell activation and differentiation^28^. These GPCRs are coupled with Gαi and inhibit the production of cAMP which is an important secondary messenger that controls cell growth, cycle and activation. In addition, the receptors also stimulate the Gβγ complex^29^. During the development of RA, the upregulated GRK2 impairs the functions of chemokine receptors, leading to imbalanced Th and Tc cell subsets, presented as the expanded Teff subgroup but diminished inhibitory Treg population. Meanwhile, the subgroup of naïve T cells was reduced. Accordingly, the proinflammtory cytokines including TNF-α and IL-1β and representative chemokine CX3CL1 were substantively expressed both in serum and synovial tissue. Of note, in CIA rats, the levels of these cytokines and chemokine were observed to be much higher in the local tissue than in the serum, aggravating the joint swelling and destruction. As we hypothesized, these changes were effectively reverted by paroxetine treatment. Bedsides reducing the infiltration of T cells, paroxetine may also modulate the function of resident cells like synoviocytes to attenuate inflammation response as we detected through H&E analysis.

Our data indicate that paroxetine induces T cells immune tolerance by reducing the population of CD4^+^ and CD8^+^ effector T cells and promoting the differentiation of Threg cells, leading to the restoration of immunity homeostasis, and as a result, it substantially prevent the progress of RA. Researchers have reported that Type II collagen (CII) could induce peripheral immune tolerance via the generation of CD8^+^ Treg cells when CII is injected into the anterior chamber (AC) of the eye^30^,^31^. Whether paroxetine and CII treatment shares common mechanisms need to be elucidated.

Paroxetine is a well-known antidepressant so it may produce psychological side effects if applied for RA treatment in clinic. Several new selective GRK2 inhibitors such as GSK180736A, Takeda103A, 12n (CCG-224406), etc. have been synthesized and/or are in clinical trials^32^. More specific and potent GRK2 activity modulators are considered to be the direction of drug development, as off-target side effects are less likely with these compounds.

Many anti-arthritis drugs are available in clinic such as non-steroidal anti-inflammatory drugs (NSAIDs), disease-modifying antirheumatic drugs (DMARDs) and biological drugs. These treatments inhibit or neutralize the production of cytokines or generally suppress the immune system. However, blocking specific immunological pathways by these violent treatments may be simultaneously successful and detrimental^33^. Although a majority of patients find relief from their RA symptoms on these regimens, they often have severe side effects associated with their gastrointestinal tracts and are more prone to acquire infections requiring hospitalization^27^. Medications developed for maintaining the immunologic equilibrium. such as GRK2 inhibitors, will be the novel trends in RA treatment that could avoid the adverse side effects that are common with current treatment options.

## Methods

### Animals

This study was approved by the Ethical Committee on Animal Research at the Institute of Clinical Pharmacology, Anhui Medical University. The methods applied in this study were carried out in accordance with the approved guidelines and regulations. Male Wistar rats aged 6 to 8 weeks, with the weight of 180 g ± 20 g, were purchased from Slack Corporation (Shanghai, China). Rats were encapsulated in a sterile environment.

### Drugs and reagents

Methotrexate (MTX, 2.5 mg per tablet) was purchased from XINYI Medical Limited Company in Shanghai, China. MTX injection (0.1 g per ampoule) was purchased from Pfizer, Inc. (NY, USA). Paroxetine, with chemical name (3S,4R)-3-((Benzo[d][1,3]dioxol- 5-yloxy) methyl)-4-(4- fluorophenyl) piperidine hydrochloride was purchased from Ark Pharm, Inc. CCII was obtained from Chondrex Corporation. Enzyme-linked immune sorbent assay (ELISA) kits for IL-1β, TNF-α and CX3CL1 were the products from Raybiotech, Inc. (GA, USA). Rabbit anti-rat GRK2, rabbit anti-rat CD3 and rabbit anti-rat β-actin were purchased from Santa Cruz Biotechnology, Inc. (CA, USA), rabbit anti-rat pERK (Thr202/Tyr204) and rabbit anti-rat ERK were ordered from Cell Signaling Technology, Inc. (China). CX3CL1 cytokine was obtained from Peprotech Inc. (Jiangsu, China). PE-CD3, FITC-CD4, PE-CD25, PE-CD8a, FITC-CD8a, PE-CD44 and Regulatory T cell detection kit were procured from eBioscience, Inc. (CA, USA). PE-CD62L was purchased from BioLegend, Inc. (CA, USA). FITC-CD11b is obtained from BD Biosciences (NJ, USA). Anti-TCRγ/δ magnetic beads were the product of Miltenyi Biotec GmbH (Germany).

### The establishment of rat CIA model

The rat CIA model was induced according to the protocol described previously^34^. Briefly, CCII was dissolved in 0.01 M filtered acetic acid by grinding on ice, and the concentration of CCII reached 4 mg/ml. The CCII was emulsified thoroughly with the same volume of Freund’s incomplete adjuvant. A total of 0.2 ml emulsion was injected intradermally into the base of the tail or back at multiple sites. Seven days later, the same emulsion was made and the rats were given a booster injection. The first immunization day was defined as day 0. The clinical symptoms of the rats were evaluated every three days and the volume of the left hind paw was determined with a YLS-7A toe volume meter (The Academy of Medical Sciences, Shandong, China).

### *In vivo* drug Administration

After the onset of CIA (around d14), model rats were randomly divided into CIA-Veh, CIA-Par and CIA-MTX groups according to the clinical scores. Paroxetine (15 mg/kg/day) and MTX (0.5 mg/kg/3days) were dissolved in 0.5% CMC-Na and administered by gavage for 15 days.

### Evaluation of arthritis

The rats were inspected every three days following the established standard from the onset of secondary inflammation^35^. The global assessment, arthritis index, swollen joints count, and secondary paw swelling were recorded and analyzed.

### H&E staining and immunohistochemistric staining

The joints from different groups after treatment were fixed in 4% formaldehyde and decalcified. The samples then were imbedded into paraffin and sectioned for H&E staining and immunohistochemistric staining following the protocols reported before^36^,^37^. The pathological score of H&E staining was analyzed by a researcher blinded to the samples. The infiltrated T cell number was counted using image pro-plus (version 6.2).

### Enzyme-linked immunosorbent assay

Serum samples were extracted from peripheral blood of the rats. The homogenate of 15 mg synovial tissue was made by an electric homogenizer with 200 μl lysis buffer containing proteinase inhibitors. The expression of IL-1β, TNF-α and CX3CL1 was detected according to the instructions of the manufacturers. The plates were read under 450 nm, and each sample was duplicated.

### Synovial tissue digestion

Synovial tissue was washed with D-Hank’s solution and cut into 1–2 mm^3^ pieces, then incubated with 4 ml collagenase type II, which was dissolved in serum free DMEM, for 1hr at 37 °C. Then the digest was spun and the pellet was incubated with 0.25% pancreatin for 30 min at 37 °C. The digest was spun again and then the pellet was resuspended with PBS. Cell suspension was filtered by 200 μm nylon mesh and stained before running through a flow cytometer.

### Flow cytometry

PBMCs were isolated using density gradient centrifugation. Cells were separated into different tubes and stained with PE-CD3, FITC-CD4, PE-CD25, PE-CD8a, FITC-CD8a, PE-CD44, FITC-CD11b or PE-CD62L. Regulatory T cells were detected by a Treg kit (FITC-CD4, PE-CD25, Pecy5-Foxp3) according to the manufacturer’s directions. The samples were tested with a flow cytometer (FC500, Beckman) and the results were analyzed using Flowjo (version 7.6).

### T cell purification and migration assay

Splenic T cells were purified through MACS Anti-TCRγ/δ magnetic beads. Subsequently, T cell migration assay was performed in a Transwell plate (Costar). 5 × 10^5^ T cells were resuspended in 100 μl DMEM and plated into a cell insert with a diameter of 6.5 mm and a pore size is 5 μm. 10 μg/ml of recombinant CX3CL1 in 600 μl was added into the lower chamber of the transwell plate. 0.1, 1, 10 μM paroxetine or 0.1, 1, 10 μM MTX was administrated into the system at the same time. The plate was incubated in 37 °C and 5% CO_2_ for 4 hours. The media in the lower chamber was collected and the number of migrated T cells was counted by using a Cellometer Vision Cell Analyzer (Nexcelom Bioscience LLC., MA, USA)^38^.

### Determination of T cell vitality

The vitality of T cells was determined using CCK kit according to the instructions. 100 μl of 5 × 10^5^/ml T cells were placed to 96 well plate in serum free media. 5 mg/ml ConA was used to stimulate T cells for 48 h. Four hours before the end of the culture, 10 μl of CCK was added into individual well, then the plate was read on a microplate reader at 450 nm. Each sample was conducted in triplicate.

### Electrotransfection

Splenic T cells were purified, and pIRES2-EGFP-GRK2 plasmid or blank pIRES2-EGFP vector was transfected into T cells by using Gene Pulser Xcell™ Electroporation Systems (Bio-Rad Laboratories, Inc. CA, USA). T cells were stimulated with 2 μg/ml ConA for 24 h to reach the mid-log phase and then were resuspended in Opti-MEM without serum at the concentration of 1 × 10^6^/ml, 100 μl of the cells and plasmid (final concentration of 50 μg/ml) were added into the electroporation cuvette and were gently mixed by tapping the side of the cuvette. The cuvette was placed in the ShockPod, 2100 V, 10 ms, pulse once, then cells were immediately transferred into a 24 well plate with 500 μl full culture media. The expression of plasmid was checked after 48 h culture^39^.

### Immunofluorescence assay

The expression of GRK2 in T cells was tested by immunofluorescence assay after the electrotransfection of GRK2 plasmid or vector. Briefly, T cells were fixed in 4% formalin for 30 min at room temperature, then spun and washed with PBS twice. Cells were permeabilized by 0.1% Triton X-100 for 30 min. The cells were blocked with goat serum for 30 min at 37 °C, followed by the incubation of rabbit-anti GRK2 primary antibody overnight at 4 °C. After 3 washes with PBS, the cells were incubated with goat anti-rabbit-Alexa Fluor 488 secondary antibody for 2 h at 37 °C. The above procedures were performed in the tube. The cells were spun and the pellet smear was observed under Leica TCS SP8 confocal microscope (Leica Microsystems, Wetzlar, Germany). The fluorescence intensity of the images was quantified by using Image J (NIH).

### Western Blot

Splenic T cells were isolated from each group and applied for western blot. The primary antibody, GRK2 (1:1000), β-actin (1:5000), pERK(1:1000), ERK(1:1000) pP38 (1:1000), P38 (1:1000), pJNK (1:1000) or JNK (1:1000) was incubated overnight in 4 °C, and goat anti-rabbit secondary antibody (1:20000) was incubated for 2 h at 37 °C. The membrane was scanned on a GS-700 Imaging Densitometer (Bio-Rad, CA, USA). The image was analyzed with ImageJ software (NIH).

### Statistical Analysis

Results are shown as mean ± SD. One-way analysis of variance (ANOVA) was used to analyze the data from multiple groups, T-test was applied for the comparison between two groups. p< 0.05 were regarded as a significant difference. The data was obtained from three repeated experiments.

## Additional Information

**How to cite this article:** Wang, Q. *et al*. Paroxetine alleviates T lymphocyte activation and infiltration to joints of collagen-induced arthritis. *Sci. Rep.* **7**, 45364; doi: 10.1038/srep45364 (2017).

**Publisher's note:** Springer Nature remains neutral with regard to jurisdictional claims in published maps and institutional affiliations.
